# SRSF3-Mediated Ki67 Exon 7-Inclusion Promotes Head and Neck Squamous Cell Carcinoma Progression via Repressing AKR1C2

**DOI:** 10.3390/ijms24043872

**Published:** 2023-02-15

**Authors:** Miaomiao Liu, Can Lin, Qiwei Huang, Jun Jia, Jihua Guo, Rong Jia

**Affiliations:** 1 The State Key Laboratory Breeding Base of Basic Science of Stomatology (Hubei-MOST) & Key Laboratory of Oral Biomedicine Ministry of Education, School & Hospital of Stomatology, Wuhan University, Wuhan 430079, China; 2 RNA Institute, Wuhan University, Wuhan 430072, China; 3 State Key Laboratory of Virology and Hubei Key Laboratory of Cell Homeostasis, College of Life Science, TaiKang Center for Life and Medical Sciences, Wuhan University, Wuhan 430072, China; 4 Department of Oral and Maxillofacial Surgery, School & Hospital of Stomatology, Wuhan University, Wuhan 430079, China; 5 Department of Endodontics, School & Hospital of Stomatology, Wuhan University, Wuhan 430079, China

**Keywords:** Ki67, SRSF3, AKR1C2, head and neck squamous cell carcinoma

## Abstract

Ki67 is a well-known proliferation marker with a large size of around 350 kDa, but its biological function remains largely unknown. The roles of Ki67 in tumor prognosis are still controversial. Ki67 has two isoforms generated by alternative splicing of exon 7. The roles and regulatory mechanisms of Ki67 isoforms in tumor progression are not clear. In the present study, we surprisingly find that the increased inclusion of Ki67 exon 7, not total Ki67 expression level, was significantly associated with poor prognosis in multiple cancer types, including head and neck squamous cell carcinoma (HNSCC). Importantly, the Ki67 exon 7-included isoform is required for HNSCC cell proliferation, cell cycle progression, cell migration, and tumorigenesis. Unexpectedly, Ki67 exon 7-included isoform is positively associated with intracellular reactive oxygen species (ROS) level. Mechanically, splicing factor SRSF3 could promote exon 7 inclusion via its two exonic splicing enhancers. RNA-seq revealed that aldo-keto reductase *AKR1C2* is a novel tumor-suppressive gene targeted by Ki67 exon 7-included isoform in HNSCC cells. Our study illuminates that the inclusion of Ki67 exon 7 has important prognostic value in cancers and is essential for tumorigenesis. Our study also suggested a new SRSF3/Ki67/AKR1C2 regulatory axis during HNSCC tumor progression.

## 1. Introduction

Ki67 is a huge nuclear protein encoded by gene *MKI67* within the human genome [1]. The entire open reading frame of *MKI67* gene contains approximately 9.7 kb and encodes a large protein of 350 kDa. [2]. Ki67 is expressed in the G1, S, and G2 phases of the cell cycle other than the G0 phase, and mainly accumulated in the S phase [3,4]. For a long time, the function of Ki67 remains unclear [5]. Recently, Ki67 is reported to be involved in keeping mitotic chromosomes apart [6], chromosome mobility [7], and organizing heterochromatin organization [8]. However, the biological functions of Ki67 remain largely unknown.

Ki67 is a well-known proliferation marker for many types of cancers [9]. Ki67 expression evaluated by the immunohistochemical staining can be used as a prognostic and prediction target in some types of cancer. However, many controversies and debates exist regarding the roles of Ki67 in tumor prognosis [10,11], which limits the clinical application of Ki67. For example, the key issue of Ki67 in the diagnostic assay of breast cancer is that the analytical validity of Ki67 expression evaluated by immunohistochemical staining needs to be improved [10,12]. Alternative Ki67 detection methods may provide new ways to improve the clinical application of Ki67.

Alternative splicing (AS) of pre-mRNA is universal in human multi-exon genes to produce multiple isoforms from a single gene. Alternative splicing greatly improves the coding potential of the human genome. However, alternative splicing dysregulation often leads to diseases, including cancer [13,14]. Notably, Ki67 exon 7 is an alternative exon. Full-length transcript with exon 7 encodes a 350 kDa protein, while transcript without exon 7 encodes a short protein with the molecular weight of 320 kDa [15,16]. Evaluation of exon 7 alternative splicing may provide a brand new method to elucidate the roles of Ki67 in tumorigenesis. So far, the roles and regulatory mechanisms of Ki67 exon 7 alternative splicing in tumorigenesis remain to be determined. Alternative splicing is mainly regulated by splicing factors. SRSF3 is a member of the SR family that has been identified as an oncogene [17] and is frequently overexpressed in many types of cancers, including HNSCC [18,19]. In this study, we found that several splicing factors are responsible for the alternative splicing of Ki67 exon 7, and SRSF3 can promote exon 7 inclusion.

Intracellular reactive oxygen species (ROS) could activate a variety of signaling networks and regulate numerous cellular processes, including proliferation [20]. Cancer cells take advantage of elevated ROS to support their proliferation [21]. Notably, periodic oscillations in the cellular redox environment could regulate cell-cycle progression [22]. The amount of reactive oxygen species increases during G2 and M phases [23]. Ki67 expression is also closely associated with cell cycle progression, but it is unknown whether there is a relationship between Ki67 and ROS.

In the present study, we analyzed the prognostic and biological roles of Ki67 exon 7 inclusion in cancers. We explored the regulatory mechanisms of alternative splicing of Ki67 exon 7, especially the key motif for exon 7 inclusion. The downstream targets of Ki67 exon 7-included full-length isoform were explored by RNA-seq analysis.

## 2. Results

### 2.1. The Inclusion Level of Ki67 Exon 7 Is Superior to Ki67 Total Transcription Level in Overall Survival Prediction of Multiple Types of Cancer

**Figure 1:**
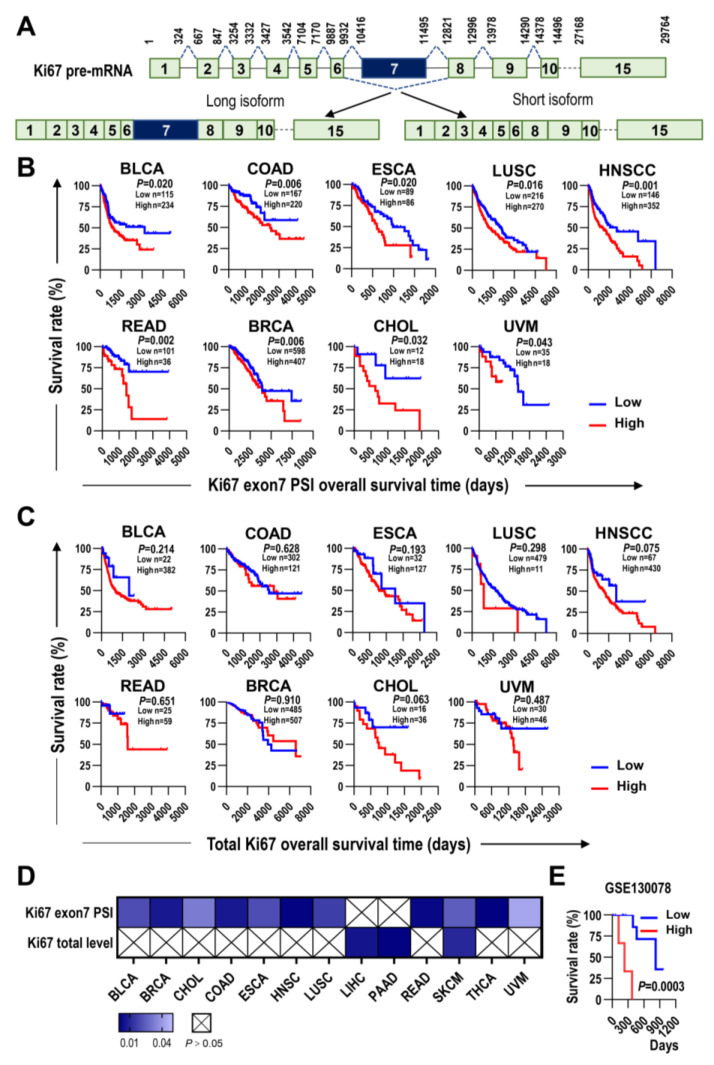
The inclusion level of Ki67 exon 7 is superior to Ki67 total transcription level in overall survival prediction of multiple types of cancer. (**A**) Schematic diagram of the alternative splicing of human Ki67 exon 7. Exon 7-included or -excluded transcripts encode long or short isoforms of Ki67, respectively. The boxes represent exons, and the lines represent introns in the pre-mRNA. The numbers above the exons and introns are the nucleotide positions in pre-mRNA. Dashed lines above or below introns indicate the RNA splicing direction. (**B**,**C**) Kaplan–Meier survival curves for overall survival (OS) based on the inclusion levels (PSI value) of Ki67 exon 7 (**B**) and the total Ki67 expression levels (**C**) in nine types of cancer (RNA-seq data, TCGA). (**D**) The heat map shows the summary of OS-related *p*-values of Ki67 exon 7 PSI and Ki67 total expression level in TCGA database. (**E**) Kaplan–Meier overall survival curves of re-analyzing Ki67 exon 7 alternative splicing in GSE130078 cohort. The *p* values were calculated using the log-rank test. BLCA—bladder urothelial cancer; COAD—colon adenocarcinoma; ESCA—esophageal carcinoma; LUSC—lung squamous cell carcinoma; HNSCC—head and neck squamous cell carcinoma; READ—rectum adenocarcinoma; BRCA—breast carcinoma; CHOL—cholangiocarcinoma; UVM—uveal melanoma.

Alternative splicing of Ki67 exon 7 produces two isoforms, the long full-length isoform with exon 7 (Ki67-F) and the short isoform without exon 7 (Ki67-Δ7) (Figure 1A). Pan-cancer analysis showed that the alternative splicing of Ki67 exon 7 could be widely detected in tumor tissues and normal tissues in The Cancer Genome Atlas (TCGA) database. Patients with the higher inclusion levels of Ki67 exon 7 showed significantly poorer overall survival rate in bladder cancer (*p* = 0.02), colon cancer (*p* = 0.006), head and neck squamous cell carcinoma (*p* = 0.001), esophageal cancer (*p* = 0.02), lung cancer (*p* = 0.016), rectal cancer (*p* = 0.002), breast cancer (*p* = 0.006), cholangiocarcinoma (*p* = 0.032), and uveal melanoma (*p* = 0.043) (Figure 1B,D). In contrast to Ki67 exon 7, the total expression level (or transcription level) of Ki67 was not significantly associated with patients’ overall survival in these cancers (Figure 1C,D). In addition, the expression levels of Ki67 exon 7-including splicing variant are significantly associated with patients’ overall survival in only three types of cancer, including colon cancer (*p* = 0.04), rectal cancer (*p* = 0.006), and lung cancer (*p* = 0.02) (Figure S1). In addition, we analyzed GSE130078 dataset, a study on esophageal carcinoma from the Gene Expression Omnibus (GEO) database [24] and confirmed that higher Ki67 exon 7 inclusion was significantly correlated with the worse patients’ overall survival (Figure 1E). These results suggested that the inclusion level of Ki67 exon 7 may represent a more attractive potential prognostic marker than the expression levels of total Ki67 or Ki67 exon 7-including splicing variant. 

### 2.2. Ki67 Exon 7 Inclusion Is Required for HNSCC Cell Proliferation, Cell Cycle Progression, and Cell Migration

**Figure 2:**
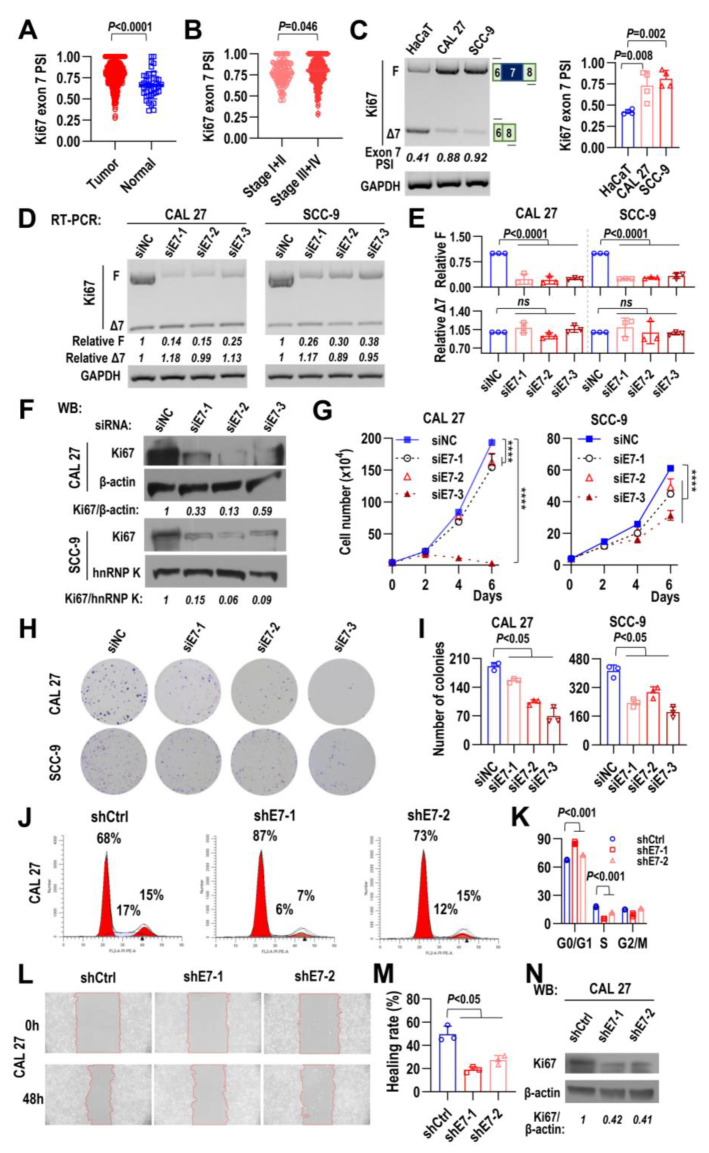
Ki67 exon 7 inclusion is required for HNSCC cell proliferation, cell cycle progression, and cell migration. (**A**) Comparison of the inclusion level of Ki67 exon 7 (PSI) between normal and tumor tissues in TCGA HNSCC dataset. (**B**) Comparison of Ki67 exon 7 PSI between patients in stage I+II and those in stage III + IV from TCGA HNSCC dataset. (**C**) Alternative splicing of Ki67 exon 7 in HaCaT (normal), CAL 27, and SCC-9 (OSCC cancer) cell lines was analyzed by RT-PCR. GAPDH served as a loading control. Diagrams on the right show the structures of Ki67 exon 7-spliced products. The histogram represents the quantification result. PSI is calculated as F/(F + Δ7). (**D**–**I**) CAL 27 and SCC-9 cells were treated with siE7s (Ki67 exon 7 targeted siRNAs) or siNC (nonspecific siRNA). (**D**) The knockdown efficiency of full-length Ki67 with exon 7 in oral cancer cells was analyzed by RT-PCR. GAPDH served as a loading control. (**E**) The histograms summarize the expression levels of Ki67 full-length isoform with or without exon 7. ns represents *p* > 0.05. (**F**) Western blot results showed the Ki67 protein levels, β-actin and hnRNP K served as loading controls. (**G**) Proliferation curves of CAL 27 and SCC-9 cells treated with siE7s or siNC. CAL 27 cells (5 × 10^4^ cells/well) or SCC-9 cells (4 × 10^4^ cells/well) were seeded into 24-well cell culture plates and transfected by siE7s and siNC on Day 0. Cells were passed and transfected again on Day 2. Cell numbers were counted on Days 2, 4, and 6. (**H**,**I**) One thousand cells treated by siE7s and siNC were seeded into 6-well cell culture plates and cultured at 37 °C for 10 days. Representative images and quantification of colony formation assay of CAL 27 and SCC-9 cells treated with siE7s or siNC. (**J**,**K**) Cell cycle analysis of CAL 27 cells with stable shE7s or shCtrl expression. Representative experiments are shown, the histogram summarizes the statistical analysis of cell cycle experiments. (**L**–**N**) Representative microphotographs of wounding healing assay of CAL 27 cells with stable shE7s (Ki67 exon 7 targeted shRNAs) or shCtrl expression. Wound healings were photographed at 0 h and 48 h. The histogram displays the summary and statistical analysis. Western blot displays the knockdown efficiency of Ki67 protein in CAL 27 cells. β-actin served as a loading control. All data are mean ± SD, *n* = 3. **** *p* < 0.0001.

Next, we explored the roles of Ki67 exon 7 inclusion in head and neck squamous cell carcinoma in the TCGA HNSCC cohort. We found that the inclusion levels of Ki67 exon 7 (PSI) of tumor tissues were significantly higher than those of normal tissues (Figure 2A). Patients at clinical-pathological stages III and IV had higher inclusion levels of Ki67 exon 7 than those at stages I and II (Figure 2B). In contrast to exon 7, there is no significant difference of Ki67 total transcription levels between patients at clinical-pathological stages III and IV and those at stages I and II (Figure S2). In addition, the Cox proportional hazards model showed Ki67 exon 7 PSI value was the independent prognostic factor of the TCGA HNSCC cohort (Table S1). In addition, HNSCC cells (CAL 27 and SCC-9) expressed significantly more full-length Ki67 isoform (Ki67-F) and less exon 7-skipped isoform (Ki67-Δ7) than HaCaT cell, an immortalized and non-tumorigenic human keratinocyte (Figure 2C). Then, we applied Ki67 exon 7-targeted siRNA to specifically down-regulate full-length Ki67 isoform (Ki67-F) without effects on exon 7-skipped isoform (Ki67-Δ7) expression in two HNSCC cell lines (Figure 2D,E). Western blot results showed Ki67 protein level significantly decreased (Figure 2F). Notably, it is almost impossible to distinguish Ki67-F protein (350 kDa) from Ki67-Δ7 protein (320 kDa) by Western blot. Downregulation of Ki67-F isoform significantly reduced cell growth (Figure 2G) and colony formation (Figure 2H,I) in both CAL 27 and SCC-9 cells. Cell cycle analysis showed that the downregulation of Ki67-F isoform significantly increased the proportion of cells in the G0/G1 phase, and decreased the proportion of cells in the S phase compared with the control in CAL 27 cells (Figure 2J,K). Further, we explored the effect of Ki67 exon 7 on the migration ability of HNSCC cells. Wound-healing migration assay showed that CAL 27 cells with stable Ki67 exon 7 downregulation migrated significantly more slowly than control group (Figure 2L–N). These results suggested that Ki67 exon 7 inclusion is required for cell proliferation, cell cycle progression, and cell migration in HNSCC cells.

### 2.3. Ki67 Exon 7 Is Essential for Tumorigenicity and Of HNSCC Cells In Vivo

**Figure 3:**
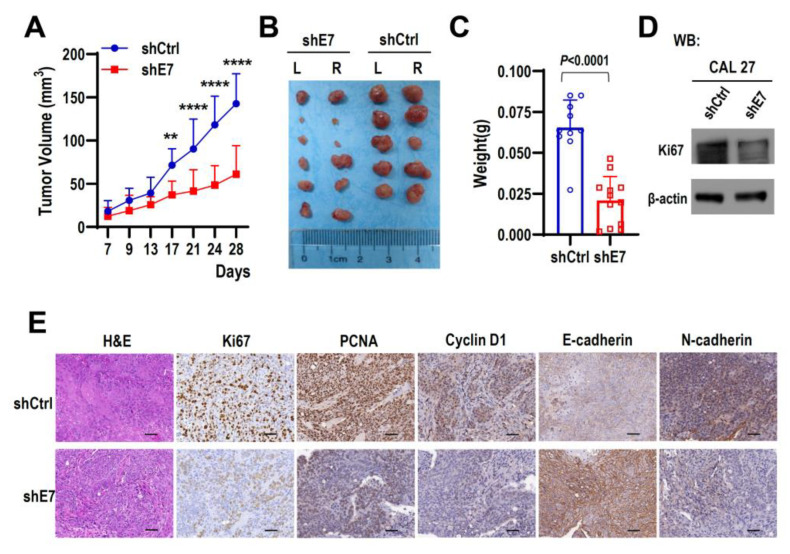
Ki67 exon 7 is essential for tumorigenicity of HNSCC cells in vivo. (**A**) CAL 27 cells with stable shE7 or shCtrl expression were injected into both sides of the flank region of BALB/c nude mice. Tumor volumes were measured on the indicated days. ** *p* < 0.01, **** *p* < 0.0001. Data are mean ± SD, *n* (shE7) = 6, *n* (shCtrl) = 5. (**B**,**C**) Tumors were dissected out and weighed on Day 28. The boxplot represents the tumor weight in two groups of mice on Day 28. (**D**) The total protein samples of cells were collected at the injection time. Western blot displayed the knockdown efficiency of Ki67 protein, β-actin served as a loading control. (**E**) Representative images of HE and immunohistochemical staining of Ki67, PCNA, cyclin D1, E-cadherin, and N-cadherin in tumors dissected from nude mice. The scale bar represents 50 μm.

Next, to test the role of Ki67 exon 7 inclusion during tumorigenesis in vivo, 1 × 10^6^ CAL 27 cells stably expressing shE7 or shCtrl shRNA were injected subcutaneously into both sides of the flank region of BALB/c nude mice. Ki67 exon 7-targeted knockdown drastically reduced tumor growth rate and tumor volumes compared with the control group (Figure 3A–C). The knockdown efficiency of Ki67 expression levels were confirmed in cultured cancer cells in vitro (Figure 3D) and tumors in vivo (Figure 3E). Moreover, immunohistochemical staining showed that tumor with Ki67 exon 7 knockdown showed reduced expression of PCNA (cell proliferation marker), Cyclin D1 (cell cycle marker), and N-cadherin (epithelial to mesenchymal transition, EMT, marker), but increased expression of E-cadherin (EMT marker). These results are correlated with reduced tumor growth and volumes induced by Ki67 exon 7 knockdown. These results indicated that Ki67 exon 7 is important in the tumorigenesis of HNSCC in vivo.

### 2.4. Full-Length Ki67 with Exon 7 Promotes Cell Proliferation and Colony Formation

**Figure 4:**
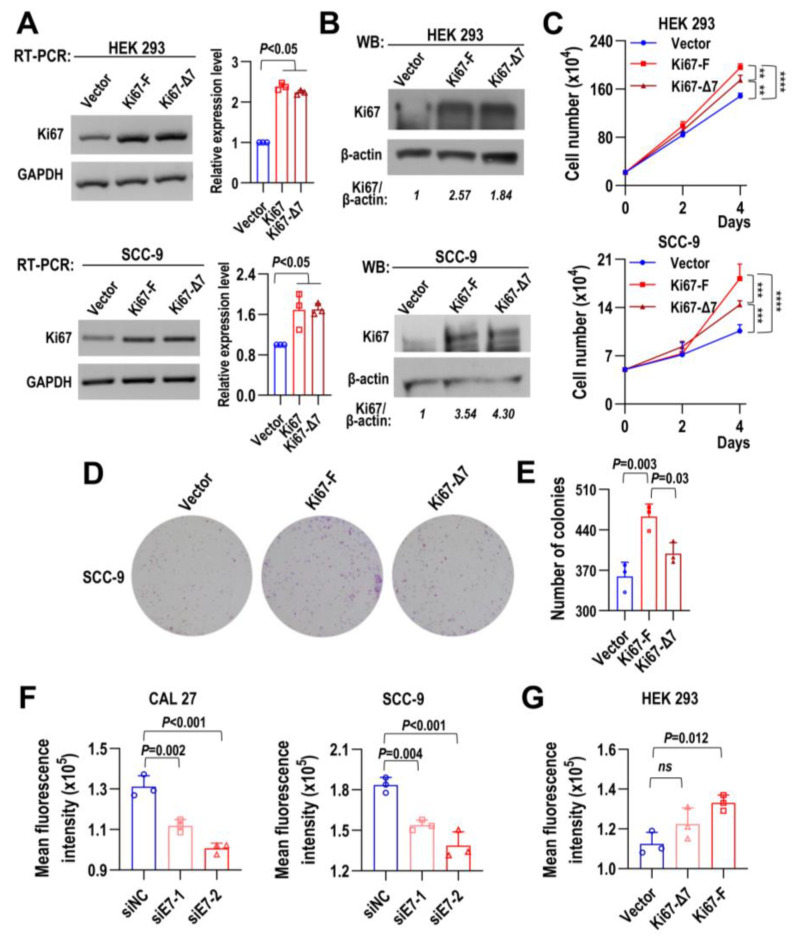
Full-length Ki67 with exon 7 promotes HNSCC cell proliferation, colony formation, and intracellular ROS production. HEK 293 and SCC-9 cells were stably overexpressed with Ki67 full-length isoform (Ki67-F), Ki67 without exon 7 isoform (Ki67-Δ7), and control vector (Vector). (**A**) The overexpression efficiency of Ki67-F and Ki67-Δ7 in both HEK 293 and SCC-9 cells were demonstrated by RT-PCR. GAPDH served as a loading control. The histograms summarize the statistical analysis. (**B**) Western blot confirmed the overexpression of Ki67-F and Ki67-Δ7 in both HEK 293 and SCC-9 cells. β-actin served as a loading control. (**C**) Proliferation curves of HEK 293 and SCC-9 cells with Ki67-F and Ki67-Δ7 stably overexpression. HEK 293 cells (22 × 10^4^ cells/well) or SCC-9 cells (5 × 10^4^ cells/well) were seeded into 24-well cell culture plates. Cells were passed on Day 2. Cell numbers were counted on Days 2 and 4. (**D**,**E**) Effect of Ki67-F and Ki67-Δ7 overexpression on the clonogenic ability of SCC-9 cells. One thousand cells were seeded in each well of 6-well cell culture plates and cultured for eight days. Representative images are shown, and the histogram summarizes the numbers and statistical analysis of colonies. (**F**,**G**) The intracellular ROS production levels of cells. CAL 27 and SCC-9 cells were treated with siE7s and siNC twice as before, and HEK 293 cells were overexpressed with Ki67-F, Ki67-Δ7, and Vector. Cell intracellular ROS level was measured by flow cytometry. The histograms summarize the mean fluorescence intensity. All data are mean ± SD, *n* = 3. ** *p* < 0.01, *** *p* < 0.001, **** *p* < 0.0001.

In contrast to the knockdown of Ki67 full-length isoform with exon 7, stable overexpression of Ki67-F significantly promoted cell proliferation in both non-tumorigenic HEK 293 cells and HNSCC cell line SCC-9 compared with Ki67-Δ7 overexpression and vector control (Figure 4A–C), as well as colony formation in SCC-9 cells (Figure 4D,E). These results indicated that exon 7 is essential for Ki67 to promote cell proliferation.

### 2.5. Full-Length Ki67 with Exon 7 Increases Intracellular ROS Production

It has been reported that Ki67 expression is tightly linked to cell cycle, and intracellular ROS is required for cell cycle progression. We hypothesized that Ki67 might be associated with cellular ROS production. Indeed, we found that Ki67 exon 7-targeted knockdown significantly reduced ROS production, while overexpression of Ki67-F isoform promoted ROS production (Figure 4F,G). Moreover, CAL 27 cells treated with both siE7 siRNA and ROS inhibitor N-Acetylcysteine (NAC) showed significantly increased G0/G1 phase than those treated with siE7 siRNA or NAC alone (Figure S3). These results suggested that Ki67 exon 7-included full-length isoform can promote intracellular ROS production to facilitate cell cycle progression and cell proliferation.

### 2.6. HNRNPC2 and SRSF3 Are Responsible for Ki67 Exon 7 Inclusion

**Figure 5:**
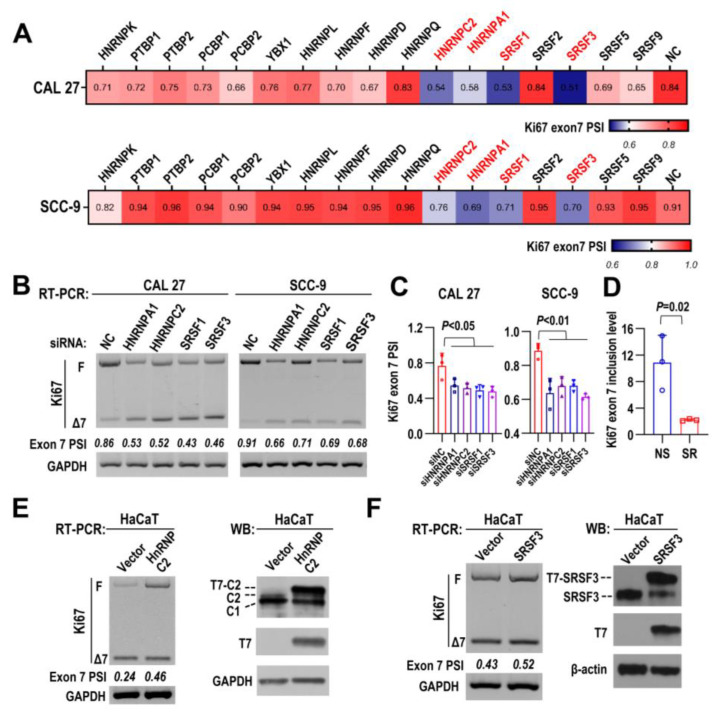
HNRNPC2 and SRSF3 are responsible for *Ki67* exon 7 inclusion. (**A**) The specific siRNAs of multiple splicing factors and nonspecific control siRNA (NC) were transfected into CAL 27 and SCC-9 cells. The heatmaps displayed Ki67 exon 7 PSI values of CAL 27 and SCC-9 cells after siRNAs treatment. (**B**,**C**) RT-PCR results of alternative splicing of Ki67 exon 7 in CAL 27 and SCC-9 cells with HNRNPA1, HNRNPC2, SRSF1, and SRSF3 knockdown. PSI is calculated as F/(F + Δ7). The histograms show the statistical analysis. Data are mean ± SD, *n* = 3. (**D**) Comparison of the Ki67 exon 7 inclusion levels in SRSF3 knockdown (SR) or control (NS) groups from GSE22149 dataset which used a SpliceArray to detect the targets of SRSF3. (**E**,**F**) HaCaT cells were stably transfected with hnRNPC2 (**E**) or SRSF3 (**F**) expression lentivirus. Alternative splicing of Ki67 exon 7 was detected by RT-PCR and GAPDH served as the loading control. Western blot displayed the overexpression efficiency of HNRNPC2 and SRSF3, GAPDH and β-actin served as loading controls.

Next, to understand the regulatory mechanisms of the alternative splicing of Ki67 exon 7, we screened 17 splicing factors in both CAL 27 and SCC-9 cells by RNAi, and the alternative splicing of Ki67 exon 7 was analyzed by RT-PCR (Figure S4). The results were summarized in Figure 5A. SRSF3, HNRNPC2, HNRNPA1, and SRSF1 were the four splicing factors whose knockdown most significantly decreased Ki67 exon 7 inclusion level (Figure 5B,C and Figure S4). We also re-analyzed a previous dataset (GSE22149), which applied a SpliceArray to analyze genome-wide splicing targets of SRSF3 in human osteosarcoma U2OS cells [25]. Ki67 exon 7 inclusion level was also significantly reduced upon SRSF3 knockdown in U2OS cells (Figure 5D). However, only HNRNPC2 or SRSF3 overexpression significantly increased Ki67 exon 7 inclusion in HaCaT cells, which has relatively low inclusion level of exon 7 (Figure 5E,F). These results demonstrated that SRSF3 and HNRNPC2 can promote Ki67 exon 7 inclusion.

### 2.7. Splicing Factor SRSF3 Interacts with Motifs in Ki67 Exon 7

**Figure 6:**
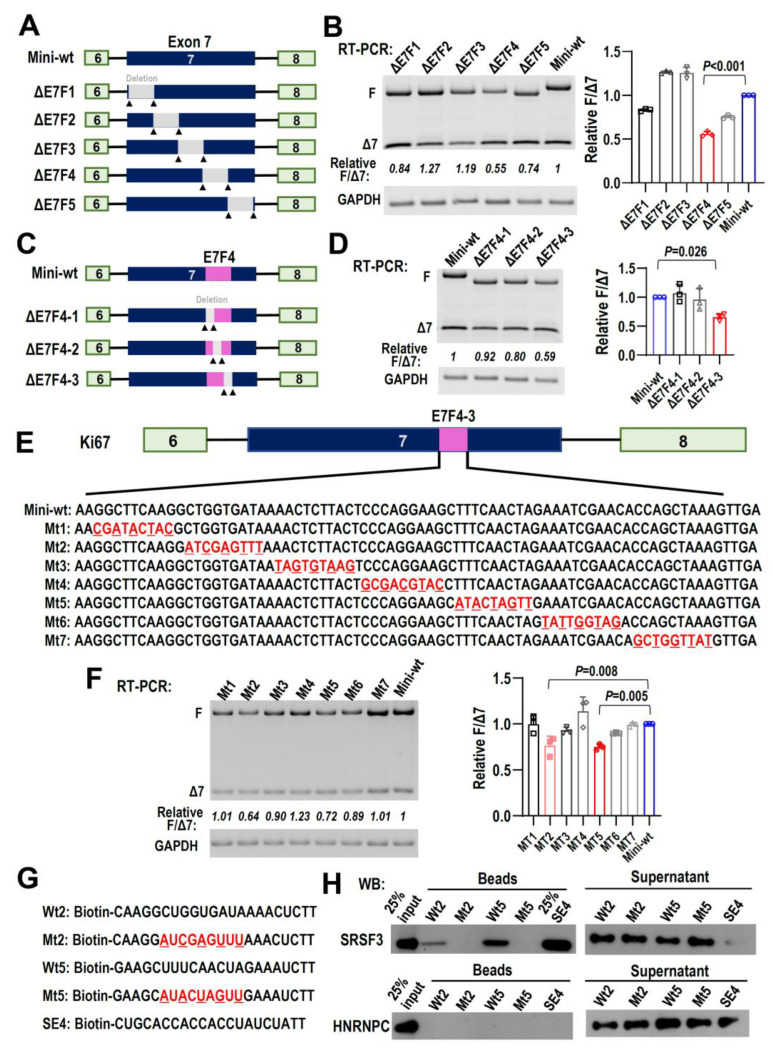
Splicing factor SRSF3 interacts with motifs in Ki67 exon 7. (**A**) Schematic diagrams of Ki67 exon 7 minigene and deletion positions in exon 7. The boxes represent exons and lines represent introns. (**B**) Five truncated minigenes ΔE7F1 to ΔE7F5 were transfected into CAL 27 cells. The alternative splicing of Ki67 exon 7 was analyzed by RT-PCR. (**C**) Schematic diagrams of Ki67 exon 7 minigene and deletion positions in fragment E7F4. (**D**) Three truncated minigenes ΔE7F4-1 to ΔE7F4-3 were transfected into CAL 27 cells. The alternative splicing of Ki67 exon 7 was analyzed by RT-PCR. (**E,F**) Point mutations in E7F4-3 region were constructed and mutated minigenes MT1 to MT7 were transfected into CAL 27 cells. The alternative splicing of Ki67 exon 7 was analyzed by RT-PCR. All the histograms display the statistical analysis. All data are mean ± SD, *n* = 3. (**G**) Biotinylated oligo RNAs were synthesized to perform the RNA pull-down assay, including wild-type (wt2 and wt5), mutant motifs (mt2 and mt5), and SE4 served as an SRSF3 positive binding control. (**H**) Biotinylated oligo RNAs were incubated with CAL 27 cell total cellular extract. Proteins binding on beads were separated in SDS-PAGE gel and blotted with SRSF3 and HNRNPC antibodies. CAL 27 cell total cellular extract was used as input. The total extracts in the supernatant after pull-down were used as the loading control.

To further explore the mechanisms that regulate alternative splicing of Ki67 exon 7, we constructed a minigene to mimic alternative splicing of exon 7. Serial deletions of exon 7 sequence and splicing analysis revealed that the deletion of the sequence in the middle of exon 7 (corresponding to ΔE7F4 deletion minigene) led to the most significant exon 7 exclusion (Figure 6A,B). Further deletion analysis of this sequence revealed that the key regulatory motif for exon 7 inclusion was located in a fragment of around 70 bp (Figure 6C,D, corresponding to ΔE7F4-3 deletion minigene). Screening analysis of this short region by point mutation showed that mutation of two motifs, mt2 and mt5, whose corresponding wide-type RNA sequences were “CUGGUGAUA” (wt2) and “UUUCAACUA” (wt5), respectively, are essential for exon 7 inclusion (Figure 6E,F). Interestingly, SRSF3, not HNRNPC2, can bind to these two motifs in an in vitro pull-down assay (Figure 6G,H). Mutants of these two motifs could not bind SRSF3 or HNRNPC2. Notably, wt5 motif showed the stronger binding capacity to SRSF3 than wt2 motif (Figure 6H). Taken together, these results showed that SRSF3 may promote exon 7 inclusion by interacting with motifs “CUGGUGAUA” and “UUUCAACUA”, two exonic splicing enhancers.

### 2.8. Cellular Targets of Ki67 Exon 7-Included Isoform in HNSCC Cells

**Figure 7:**
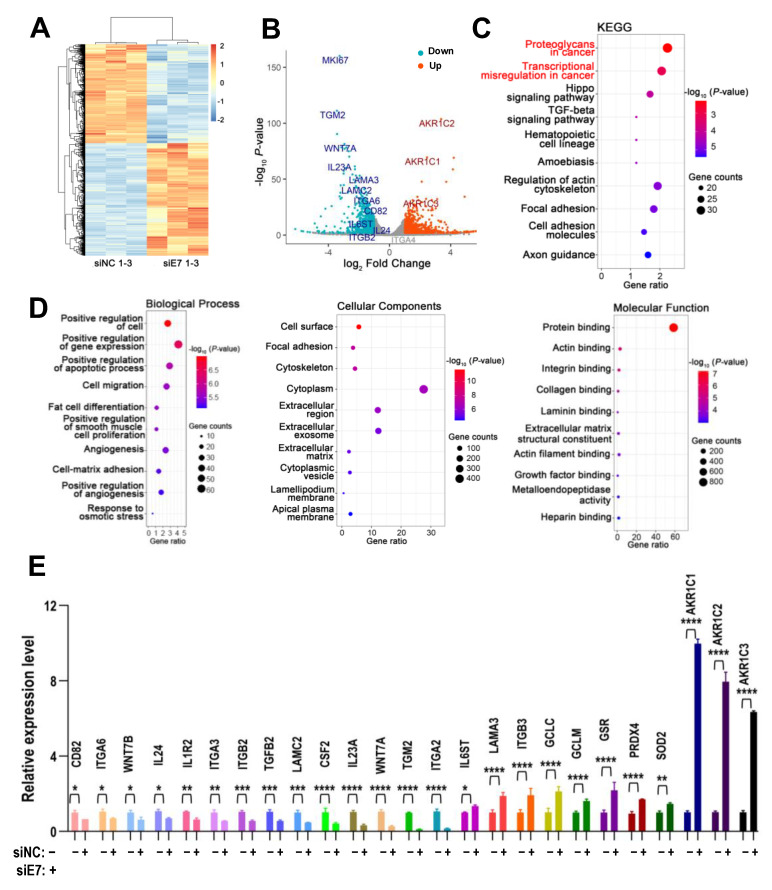
Cellular targets of Ki67 exon 7-included isoform in HNSCC cells. (**A**) The heatmap of the differentially expressed genes (DEGs) in SCC-9 cells with siE7 and siNC treated identified by RNA-seq. (**B**) The volcano plot of DEGs, each dot represents a gene. The red color denotes upregulated genes and the fluorescent blue color denotes downregulated genes. (**C**) KEGG pathways analysis of DEGs. (**D**) GO analysis of DEGs, including biological processes (BP), cell component (CC), and molecular function (MF). All the KEGG and GO graphs show the top 10 significant terms. The x-axis is the percent of DEGs enriched in the KEGG pathways or GO terms, and the y-axis is the corresponding KEGG pathway or GO term names. The bubble size represents the number of differentially expressed genes, and the bubble color represents the *p*-value. (**E**) Twenty-five DEGs were verified by qRT-PCR. * *p* < 0.05, ** *p* < 0.01, *** *p* < 0.001, **** *p* < 0.0001.

To understand the molecular mechanisms of Ki67 exon 7-included isoform in cancer, we analyzed cellular targets of Ki67 exon 7-included isoform by RNA-seq in SCC-9 cells treated with siRNA against Ki67 exon 7 or control siRNA. The RNA-seq data displayed 1707 differential expressed genes (DEGs), among which 798 genes were down-regulated and 909 genes were up-regulated with the |log_2_FC| > 1 and adjusted *p*-value < 0.05 (Figure 7A,B). KEGG analysis showed that DEGs were enriched in pathways related to cancer, including “proteoglycans in cancer” and “transcriptional misregulation in cancer”. Another two enriched pathways, “Hippo signaling pathway” and “TGF-beta signaling pathway”, also play roles in tumorigenesis (Figure 7C). Moreover, DEGs were associated with biological processes related to cancer, including “cell migration”, “positive regulation of apoptotic process”, and “angiogenesis” in GO biological processes analysis (Figure 7D). Twenty-five DEGs were further verified by qRT-PCR (Figure 7E). These results indicated that inclusion of Ki67 exon 7 was important for the cancer-related pathways and biological processes.

### 2.9. Ki67 Exon 7-Included Isoform Promotes Tumorigenesis by Inhibiting AKR1C2 Expression

**Figure 8:**
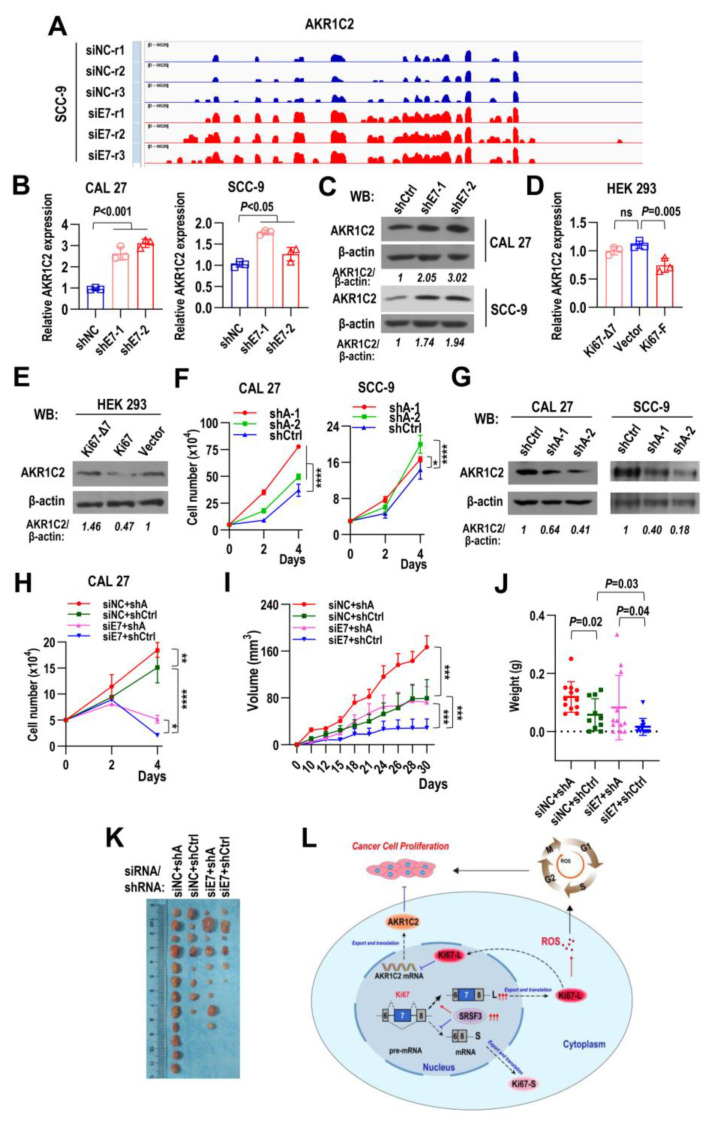
Ki67 exon 7-included isoform promotes tumorigenesis by inhibiting AKR1C2. (**A**) The AKR1C2 mRNA expression level of SCC-9 cells treated with siE7 and siNC was analyzed by IGV software. (**B**,**C**) AKR1C2 mRNA and protein expression levels in CAL 27 and SCC-9 cells with stable shE7s or shCtrl expression were analyzed by qRT-PCR and Western blot, β-actin served as a loading control. (**D**,**E**) AKR1C2 mRNA and protein levels in HEK 293 cells with stable Ki67-F, and Ki67-Δ7 overexpression were detected by qRT-PCR and Western blot. Vector is empty control. β-actin served as a loading control. (**F**) CAL 27 cells (5 × 10^4^ cells/well) and SCC-9 cells (3 × 10^4^ cells/well) with stable shAs or shCtrl expression were seeded into 24-well plates on Day 0. Cells were passed on Day 2. Cell numbers were counted on Days 2 and 4. Data are mean ± SD, *n* = 3. (**G**) Western blot displayed the knockdown efficiency of AKR1C2. β-actin served as a loading control. (**H**) Downregulation of AKR1C2 partially rescued cell growth inhibition induced by siRNA against Ki67 exon 7 in CAL 27 cells. CAL 27 cells with stable shAs and shCtrl expression (5 × 10^4^ cells/well) were seeded into 24-well plates, siE7 or siNC were transfected after two hours. Cells were passed on Day 2 and transfected again. Cell numbers were counted on Days 2 and 4. Data are mean ± SD, *n* = 3. (**I**) Downregulation of AKR1C2 partially rescued tumor growth inhibition induced by siRNA against Ki67 exon 7 in vivo. CAL 27 cells treated as (**H**) were inoculated into BALB/c nude mice (3.5 × 10^5^ cells per side). Tumor volumes were measured on the indicated days. (**J**,**K**) Tumors were dissected and weighed on Day 30. Data are mean ± SD, *n* = 6. (**L**) The summarized working model of findings in this study. * *p* < 0.05, ** *p* < 0.01, *** *p* < 0.001, **** *p* < 0.0001.

Of all the DEGs, AKR1C2 was the most significantly upregulated gene in RNA-seq data (Figure 7B and Figure 8A). AKR1C2 is a member of aldo-keto reductases (AKRs) family and can regulate metabolism of steroids hormone by inactivating 5α-dihydrotestosterone (5α-DHT). qRT-PCR and Western blot results confirmed that shRNA against Ki67 exon 7 significantly increased the transcriptional level of AKR1C2 in both CAL 27 and SCC-9 cells (Figure 8B,C). Meanwhile, overexpression of Ki67 exon 7-included isoform (Ki67-F), not exon 7-skipped isoform (Ki67-Δ7), decreased the transcription (Figure 8D, qRT-PCR) or protein level (Figure 8E, Western blot) of AKR1C2 expression in HEK 293 cells. Knockdown of AKR1C2 significantly promoted cell proliferation of CAL 27 and SCC-9 cells (Figure 8F,G). Importantly, stable downregulation of AKR1C2 can partially rescue the suppression of cell proliferation caused by siRNA against Ki67 exon 7 in CAL 27 cells (Figure 8H). Moreover, stable downregulation of AKR1C2 can partially rescue the suppression of tumor development caused by siRNA against Ki67 exon 7 in vivo (Figure 8I–K and Figure S5). AKR1C2 functions as a tumor suppressor during the tumorigenesis of CAL 27 in nude mice. These results suggest that Ki67 exon 7-included isoform promote tumorigenesis of HNSCC by inhibiting AKR1C2 expression.

## 3. Discussion

In this study, we demonstrated that patients with higher inclusion levels of Ki67 exon 7 had poorer overall survival rate in at least nine types of cancer. In contrast, the total Ki67 expression level is not associated with patients’ overall survival rate in these cancers. Total Ki67 protein was routinely analyzed in many cancers by immunohistochemistry to reflect the proliferation of tumor cells [9], and used as a prognostic marker in many cancers, such as breast cancer [26] and gastric cancer [27]. However, debates exist regarding the value of Ki67 in tumor prognosis. For example, Ki67 staining has limited value in the treatment decision of breast cancer [28]. Ki67 is suggested not to be used as a biomarker for breast cancer patients having 1–3 positive axillary nodes [10]. Ki67 staining level does not correlate with survival and is not a useful prognostic marker in pancreas cancer [29]. Notably, the validity of Ki67 staining is often poor between different laboratories [30] due to some factors in evaluating the scores of Ki67 staining, such as software and analytical instruments, the differences in interpretation between personnel’s judgment, and the certain heterogeneity of tumor tissues [31]. In the present study, we analyzed the inclusion level of Ki67 exon 7, which may be more quantifiable and standardizable than immunohistochemistry. Notably, the level of Ki67 exon 7 inclusion is superior to Ki67 total transcription level in survival prediction of multiple types of cancer. Exon 7-included isoform of Ki67 may be a better potential biomarker of head and neck cancer than the total expression level of Ki67. However, more researches are required to evaluate the value of the alternative splicing of Ki67 exon 7 in predicting the progression of head and neck cancer. In addition, this method requires regular reagents and equipment for the isolation and purification of tumor RNA samples, performing RT-PCR, and analyzing the inclusion of Ki67 exon 7. Therefore, this method is not that expensive, but may be somewhat labor-consuming. 

Ki67 has several isoforms [15], in which the longest full-length isoform and exon 7-skipped isoform are two major isoforms [2]. However, the expression and function of Ki67 isoforms remain largely unknown. In the present study, we discovered that exon 7-included isoform is overexpressed in cancer tissues, and correlated with patients’ survival. Knockdown of exon 7-included isoform significantly attenuated cell proliferation and tumorigenesis of cancer cells. Dysregulated alternative splicing events are frequently reported in the development and progression of HNSCC. Alternative splicing of Ki67 exon 7 may be a novel target for the treatment of HNSCC.

Splicing factors play key roles in the regulation of alternative splicing. In the present study, we discovered that SRSF3 promoted the inclusion of Ki67 exon 7 via two exonic splicing enhancers in exon 7. Splicing factor SRSF3 is an oncogene and is overexpressed in many cancers, including HNSCC [17,19]. We previously found that SRSF3 could promote the skipping of heterogeneous nuclear ribonucleoprotein L (HNRNPL) exon 7 and the expression level of oncogenic HNRNPL full-length protein in HNSCC cells [32]. Our present results reveal that Ki67 is a new target for SRSF3.

Molecular targets and associated pathways of Ki67 have been scarcely revealed to date. In this study, we applied RNA-seq and identified a series of signaling pathways and target genes regulated by Ki67 exon 7-included isoform. Among these target genes, AKR1C2 was the most significantly upregulated one. AKR1C2 is a member of the aldo-keto reductases (AKRs) family which are monomeric NAD(P)(H)-dependent oxidoreductases [33]. AKR1C2 expression is positively correlated with favorable tumor characteristics and prolongs survival time in primary breast cancer patients [34]. Elevated AKR1C2 expression also indicates a favorable prognostic and recurrence-free survival rate in papillary thyroid carcinoma [35]. Consistent with these studies, we identified that AKR1C2 is a tumor suppressor in HNSCC. Knockdown of AKR1C2 could promote HNSCC cell proliferation and rescue the retarded cell proliferation induced by siRNA against Ki67 exon 7. In addition, the reduced tumorigenesis caused by siRNA against Ki67 exon 7 in vivo could also be rescued by the knockdown of AKR1C2. Overall, our study suggested a new Ki67/AKR1C2 axis in HNSCC tumor progression. However, the underlying mechanisms of how Ki67 exon 7-included variant regulating AKR1C2 expressing remain unclear. Ki67 participates in a wide range of cellular processes and can interact with many partners [36], many of which are involved in transcription, splicing, and protein translation [8]. AKR1C2 expression is transactived by nuclear factor erythroid 2-related factor 2 (Nrf2), a transcriptional factor [37]. A research showed a strong positive correlation between Ki67 and Nrf2 expression during mouse embryonic development [38]. Therefore, Ki67 may interact with Nrf2 and promote AKR1C2 expression. Future studies are required to explore this potential molecule mechanism.

Ki67 is a well-known cell proliferation marker. However, its molecular function remains largely unknown. In the present study, we discovered that Ki67 exon 7-included full-length isoform could promote cellular ROS production. Overexpressed Ki67 exon 7-included isoform increased intracellular ROS generation, while knockdown of Ki67 exon 7-included isoform inhibited ROS generation and induced a cell cycle block in the G1 phase. Intriguingly, it has been reported that mitochondrial ROS can drive cell cycle progression and cell proliferation. Notably, ROS level varied during the cell cycle progression. Cellular ROS increased from G1 to G2/M phases [23,39,40]. Ki67 expression level changes along with cell cycle progression. Ki67 mRNA was highest in G2, whereas protein levels increase throughout the cell cycle, peaking at mitosis [3]. Our results suggested that Ki67 may regulate ROS level to fit cell cycle progression. Future researches are needed to determine the mechanisms of how Ki67 increases cellular ROS level.

## 4. Materials and Methods

### 4.1. TCGA Datasets

The gene expression data derived from FPKM (fragments per kilobase of transcript per million fragments mapped) and clinical information of cancers were downloaded from the TCGA data portal (https://portal.gdc.cancer.gov/, accessed on 1 February 2020). The FPKM expression data were converted into TPM (transcripts per million) data by R software (v3.6.3, https://www.r-project.org/) and logarithmically transformed (base 2). Percent Spliced In index (PSI) values [41] of tumor samples and adjacent normal samples were downloaded from the TCGA SpliceSeq database (http://bioinformatics.mdanderson.org/TCGASpliceSeq, accessed on 1 February 2020), in which the PSI values of Ki67 exon 7 alternative splicing were calculated by dividing the normalized read counts of variant including exon 7 by the total normalized read counts of Ki67 gene (both exon 7 inclusion and exclusion). 

### 4.2. GEO Dataset

GEO database was used to search qualified cohorts for the validation of TCGA data results. Inclusion criteria include the following: (1) the public RNA-seq data; (2) clinical information, especially survival information of patients is available; (3) sample size is greater than 10. The clinical data of qualified cohort was downloaded from GEO database (https://www.ncbi.nlm.nih.gov/geo/, accessed on 18 July 2020). The sra files were downloaded from SRA database (https://www.ncbi.nlm.nih.gov/sra, accessed on 27 February 2021) and converted to fastq files using the sratoolkit.2.10.9 (NCBI, Bethesda, MD, USA). Quality of the reads was analyzed using FastQC v0.11.8 tool (Babraham Institute, Cambridge, UK). Raw reads were quality filtered and trimmed, and then mapped to the human genome (hg38) by STAR 2.7.7a and counted by featureCounts 2.0.1 (Walter and Eliza Hall Institute, Melbourne, Australia). Analysis of differentially expressed genes (DEGs) was performed by edgeR. Alternative splicing profiles of Ki67 were analyzed by rMATS 4.1.0 (https://rnaseq-mats.sourceforge.net/).

### 4.3. Cell Culture

CAL 27, SCC-9, HEK 293 cells, and HEK 293T cells were obtained as previously described [42]. HaCaT cells were purchased from Procell Life Science (Procell, Wuhan, China). CAL 27, HEK 293, and HEK 293T cells were maintained in dulbecco’s modified eagle’s medium (DMEM) high glucose (Hyclone, Marlborough, MA, USA) supplemented with 10% fetal bovine serum (Gibco, Carlsbad, CA, USA) and 1% antibiotic-antimycotic (Invitrogen, Carlsbad, CA, USA). SCC-9 cells were cultured in a 1:1 mixture of DMEM and Ham’s F12 medium containing 10% FBS, 400 ng/mL hydrocortisone, and 1% antibiotic-antimycotic. HaCaT cells were cultured in minimum essential medium (MEM) (Procell, Wuhan, China) containing 10% FBS and 1% antibiotic-antimycotic.

### 4.4. siRNA and Transfection

Three human Ki67 exon 7 specific siRNAs were synthesized in GenePharma (Shanghai, China). The sequences are 5′-GAAGCUUUCAACUAGAAAUCG-3′ (siE7-1), 5′-GGUCUUAGUUCAGUUGAUAUC-3′ (siE7-2), and 5′-GUUCAGUUGAUAUCAACAACU-3′ (siE7-3). Nonspecific siRNA (siNC) was obtained from GenePharma (Shanghai, China). siNC was used as a control. The sequences of siRNA against splicing factors are shown in Table S2. All siRNAs were transfected into cells using the Lipofectamine 3000 Reagent (Invitrogen, Carlsbad, CA, USA) according to the manufacturer’s protocol. CAL 27 or SCC-9 cells were seeded into 12-well plates and transfected by 10 nM siRNA on Day 0. Cells were passed and transfected again on Day 2. Total protein and RNA were collected on Day 4.

### 4.5. Plasmids and Transfection

The full-length open reading frame or exon 7-excluded version of human *MKI67* gene named Ki67-F and Ki67-Δ7 were amplified from HEK 293 cells, and cloned into pLVX-IRES-Zsgreen vector at EcoRI and SpeI sites by using ClonExpress MultiS One Step Cloning Kit (Vazyme Biotech, Nanjing, China) according to the manufacturer’s instructions. Primers used are listed in Table S3. The anti-Ki67 exon 7 shRNA (shE7s) or anti-AKR1C2 shRNA (shAs) plasmids and nonspecific shRNA (shCtrl) expression plasmid were produced by Vector Builder Inc. (Guangzhou, China). The T7-SRSF3 expression plasmid was kindly provided by Dr. Zheng Zhi-Ming (National Cancer Institute, Bethesda, MD, USA). HNRNPC2 expression plasmid was constructed by cloning the T7-tagged open reading frame of HNRNPC2 gene into pLVX-IRES-puro vector (Clontech, Kusatsu, Japan) at EcoRI and NotI sites. Lentivirus was produced by co-transfecting expression plasmid with psPAX2 and pMD2.G into HEK 293T cells.

### 4.6. Minigene Assay

The minigene containing exons 6–8 and introns 6–7 of the human *MKI67* gene was amplified from HEK 293 cell genome DNA and cloned into pEGFP-N1 (Clontech) vector at EcoRI and BamHI sites. To map the essential motifs for exon 7 splicing, further deletions or mutations were introduced into minigene by overlapping PCR (Figure 6A,C,E). Primers used to construct the minigene plasmids are listed in Table S3.

### 4.7. Semiquantitative Reverse Transcription PCR (RT-PCR) and Real-Time Quantitative Reverse Transcription PCR (qRT-PCR)

Total RNA was purified using AxyPrep Multisource Total RNA Miniprep Kit (Axygen, Union City, CA, USA) according to the manufacturer’s protocol. Reverse transcription was performed using the Maxima H Minus cDNA Synthesis Master Mix (Thermo Scientific, Carlsbad, CA, USA), and then semi-quantitative PCR was performed using Green Taq Mix (Vazyme Biotech, Nanjing, China). The primers used in RT-PCR are listed in Table S4. Quantitative real-time PCR was carried out using ChamQ Universal SYBR qPCR Master Mix (Vazyme Biotech, Nanjing, China), and performed with the CFX96 real-time PCR detection system (Bio-Rad, Hercules, CA, USA). The primers used in qRT-PCR are listed in Table S5.

The band intensity of specific RT-PCR products was quantified using Image J software (NIH, Bethesda, MD, USA). The PSI values of Ki67 exon 7 alternative splicing in RT-PCR were calculated by dividing the band intensity of variant including exon 7 by the total band intensities of Ki67 gene (both exon 7 inclusion and exclusion products), namely, PSI = F/(F + Δ7). “F” represents the full-length variant of Ki67 including exon 7, and “Δ7” represents the short variant of Ki67 excluding exon 7. In this study, both RNA-seq and RT-PCR assays calculate PSI values by dividing the expression level of variant containing exon 7 by the total expression level of Ki67 exon 7 inclusion and exclusion variants. However, in principle, PSI values calculated based on read fragment counts of RNA-seq data are more accurate than those calculated based on band intensity of RT-PCR images.

### 4.8. RNA-seq

SCC-9 cells were transfected with siE7 and siNC on Day 0, cells were passed and transfected again on Day 2, and total RNA was purified on Day 4 using TRIzol Reagent (Invitrogen, Carlsbad, CA, USA) and then used for mRNA sequencing (Wuhan Seqhealth Co., Ltd. Wuhan, China). Stranded RNA sequencing library preparation used KCTM Stranded mRNA Library Prep Kit for Illumina following the manufacturer’s instruction. PCR products corresponding to 200–500 bps were enriched, quantified, and finally sequenced on Novaseq 6000 sequencer (Illumina, San Diego, CA, USA) with PE150 model. The DESeq2 software package was used to analyze differential gene expression. DEGs were analyzed by Kyoto Encyclopedia of Genes and Genomes (KEGG) and Gene Ontology (GO) analysis using the online DAVID database (www.david.niaid.nih.gov, accessed on 26 June 2022).

### 4.9. Western Blot

Total cellular protein samples were prepared by lysing cells with 2 × SDS sample buffer, and samples were denatured for 3 min at 95 °C, then separated in a 10% SDS-PAGE gel (GeneScript, Nanjing, China), followed by being transferred to a nitrocellulose membrane (Pall Corporation, Port Washington, NY, USA). Membranes were blocked with 5% skim milk and incubated with the following antibodies: anti-β-actin (Santa Cruz Biotechnology, Dallas, TX, USA, Cat# sc-47778), anti-GAPDH (Santa Cruz Biotechnology, Dallas, TX, USA, Cat# sc-47724), anti-HNRNPC (Santa Cruz Biotechnology, Dallas, TX, USA, Cat# sc-32308), anti-SRSF3 (Santa Cruz Biotechnology, Dallas, TX, USA, Cat# sc-13510), anti-Ki67 (Abcam, Cambridge, UK, Cat# ab16667), and anti-AKR1C2 (ABclonal, Wuhan, China, Cat# A1048).

### 4.10. Colony Formation

Cells with siRNA treatment were seeded at a density of 1000 cells per well in 6-well cell culture plates in triplicate and cultured at 37 °C for 8–10 days. Then cells were fixed in 4% paraformaldehyde for 15 min, followed by staining with crystal violet for 15 min at room temperature. Colonies containing more than 50 cells were regarded as positive clones. Clones were imaged under the fluorescence microscope, and the area of each colony was quantified by ImageJ software (NIH, Bethesda, MD, USA).

### 4.11. Wound-Healing Migration Assay

CAL 27 cells with stable expression of shE7 and shCtrl shRNA were seeded into 6-well cell culture plates and cultured until 95–100% confluence. An artificial scratch was created using a 200 μL pipette tip, and then cells were cultured in serum-free medium. Wound healing was photographed at 0 h and 48 h. The distances of cells migrated were calculated by ImageJ software (NIH, Bethesda, MD, USA).

### 4.12. Cell Cycle Analysis

CAL 27 cells with stable expression of shE7 and shCtrl shRNA were collected and fixed with 70% ethanol. Cells were stained with propidium iodide (PI) using a Cell Cycle Staining Kit (Multi sciences Lianke Biotech., Hangzhou, China) at room temperature for 30 min in the dark. Flow cytometric analysis was performed with CytoFLEX Flow Cytometer (Beckman, Indianapolis, IN, USA).

### 4.13. Detection of ROS

We examined the production of ROS in cells using the ROS-sensitive fluorescent dye dihydroethidium (DHE, Sigma-Aldrich, Saint Louis, MO, USA). Briefly, cells were incubated with 10 µM DHE at 37 °C in the dark for 30 min. Cells were trypsinized and rinsed twice with PBS, resuspended in staining buffer, and used immediately for FACS analysis. The mean fluorescence intensity (MFI) of DHE staining was analyzed with CytoFLEX Flow Cytometer (Beckman, Indianapolis, IN, USA) at 580 nm.

### 4.14. RNA Pull-Down Assay

Biotin-labeled Ki67 exon 7 RNA oligonucleotides wt-2 (Biotin- CAAGGCUGGUGAUAAAACUCTT, wild type), mt-2 (Biotin- CAAGGAUCGAGUUUAAACUCTT, mutations were underlined), wt-5 (Biotin-GAAGCUUUCAACUAGAAAUCTT, wild type), mt-5 (Biotin-GAAGCAUACUAGUUGAAAUCTT, mutations were underlined), and SE4 (Biotin-CUGCACCACCACCUAUCUATT, positive SRSF3 binding motif control [43]) were synthesized by Generay Biotech (Shanghai, China). The total CAL 27 cellular extract was prepared by using Pierce™ IP lysis buffer (ThermoFisher Scientific, Carlsbad, CA, USA). Five microliters biotin-labeled RNA oligonucleotide (40 μM) was blended with 100 μL Dynabeads™ MyOne™ Streptavidin T1 (ThermoFisher Scientific, Carlsbad, CA, USA) at 4 °C for 2 h. The bead-RNA complex was then incubated with total cellular extract of CAL 27 cells overnight at 4 °C. The proteins on beads were eluted by 2× SDS sample buffer.

### 4.15. Tumorigenicity in Nude Mice

Five-week-old female BALB/c nude mice were purchased from Hunan SJA Laboratory Animal Co., Ltd. (Changsha, China). All mice were housed in the SPF Animal Facility of School and Hospital of Stomatology, Wuhan University. Nude mice were anaesthetized with isoflurane inhalation and transfected CAL 27 cells were inoculated into both sides of the dorsum of nude mice. Tumor sizes were monitored every 3 to 4 days and tumor volume was calculated as (length × width^2^)·π/6. Mice were euthanized with the inhalation of carbon dioxide on a designated day, and then tumors were dissected and weighed. All animal experiments were conducted under guidelines approved by the Institutional Animal Ethics Committee, Hospital of Stomatology, Wuhan University (S07921050F and S07922030L).

### 4.16. Histological and Immunohistological Analyses

Tissues were fixed in formalin and embedded in paraffin, then sections (4 µm) were cut. Following deparaffinization with xylene, the sections were rehydrated with graded ethanol and washed with PBS, then stained with hematoxylin and eosin for histological analysis. The immunohistochemistry staining of sections was performed by using the Dako EnVision FLEX kit (Dako, Glostrup, Denmark) according to the manufacturer’s instructions as previously described [44]. Briefly, the sections were subjected to antigen retrieval in Target Retrieval Solution (Dako, Glostrup, Denmark). Endogenous peroxidase was blocked with Peroxidase-Blocking Reagent (Dako, Glostrup, Denmark). Then, the sections were incubated with primary antibody at 4 °C overnight and followed by incubation with secondary antibody FLEX/HRP (Dako, Glostrup, Denmark) at room temperatures for 30 min. Staining was developed by diaminobenzidine (DAB) substrate (Dako, Glostrup, Denmark). The stained sections were scanned by Pannoramic MIDI (3D HISTECH, Budapest, Hungary). Primary antibodies used in this study were Ki67 (Abcam, Cat# ab16667), PCNA (CST, Danvers, MA, USA, Cat# 13110), Cyclin D1 (CST, Danvers, MA, USA, Cat# 55506), E-Cadherin (CST, Danvers, MA, USA, Cat# 3195), and N-Cadherin (CST, Danvers, MA, USA, Cat# 13116).

### 4.17. Overall Survival Analysis

All the samples for survival analysis with an overall survival time of less than 10 days were excluded. Each cohort was divided into high or low groups according to the best cutoff of Ki67 exon 7 PSI values across the cohorts. The best cutoff PSI value between low and high groups was calculated by the R package “survivalROC”. The PSI value with the maximal sum of sensitivity and specificity in the receiver operating characteristic (ROC) curve is the best cutoff value. Survival analysis was performed with the log-rank test and survival curves were produced using the Kaplan–Meier method (GraphPad Prism, San Diego, CA, USA). The same grouping and survival analysis methods were applied to the overall survival analyses based on Ki67 mRNA expression levels or the expression levels of Ki67 exon 7-including splicing variant.

### 4.18. Statistical Analysis

All statistical comparisons of means were performed with Student’s *t*-test or one-way ANOVA test (GraphPad Prism). Mann–Whitney test was used to analyze the Ki67 exon 7 inclusion levels between tumor and normal groups in cells and tissues (GraphPad Prism, San Diego, CA, USA). Univariate and multivariate cox regression were used to indicate whether Ki67 exon 7 PSI was an independent prognostic factor in the TCGA HNSCC dataset (SPSS, Chicago, IL, USA). 

## 5. Conclusions

In summary, we demonstrate that the inclusion level of Ki67 exon 7 is a novel potential valuable predictor of the patients’ overall survival in numerous cancer types, and is associated with the progression and prognosis of HNSCC. Ki67 exon 7-inclusion is essential for cell proliferation, cell cycle progression, cell migration, and tumorigenesis. Oncogenic splicing factor SRSF3 directly promotes Ki67 exon 7-inclusion via two exonic splicing enhancers in exon 7. Mechanically, Ki67 can increase cellular ROS levels and repress the expression of aldo-keto reductase AKR1C2, which is a novel target of Ki67. Our study suggests a new SRSF3/Ki67/AKR1C2 axis in HNSCC tumor progression (Figure 8L).

## Data Availability

The RNA-Seq data have been deposited in the GEO database (GSE 213022).

## Supplementary Materials

The supporting information can be downloaded at: https://www.mdpi.com/article/10.3390/ijms24043872/s1. References [32,43,45,46,47,48,49,50,51] are cited in Supplementary Materials.
