# Supplementary material for: SRSF3-Mediated Ki67 Exon 7-Inclusion Promotes Head and Neck Squamous Cell Carcinoma Progression via Repressing AKR1C2
Source: Int J Mol Sci. 2023 Feb 15;24(4):3872. doi: 10.3390/ijms24043872 (PMC9959251; doi:10.3390/ijms24043872)

Figure S1. Kaplan-Meier survival curves for overall survival (OS) based on the RSEM values of the Ki67 exon 7-including splicing variant in nine types of cancer from the TSVdb database (www.tsvdb.com/instruction.html).

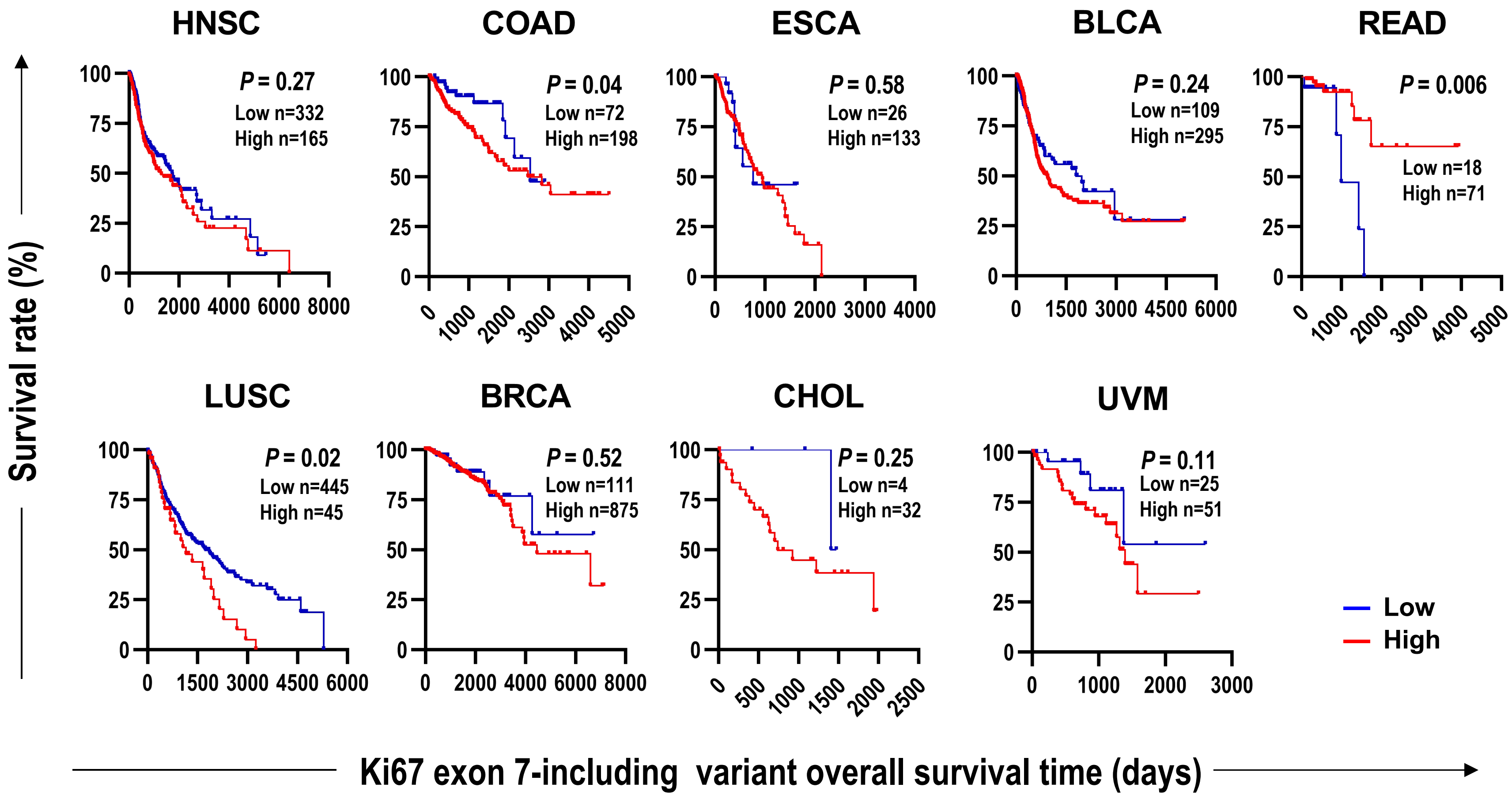

Figure S2. Total Ki67 expression levels in patients at pathological stages I+II and III+IV from the TCGA HNSCC dataset.

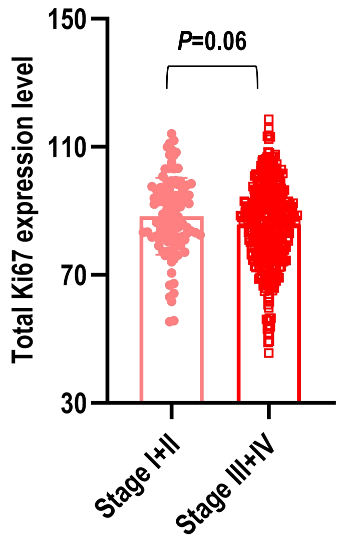

Figure S3. Cell cycle analysis of CAL 27 cells treated with both siE7 and NAC. CAL 27 cells were transfected with siNC or siE7 for 24h, and then treated with or without 10 mM NAC for another 24h. (**A**) Representative results of cell cycle. (**B**) The histogram summarizes the statistical analysis of the proportion of cells in different phases in the cell cycle. Data are mean ± SD, n = 3. ns: *P*>0.05; **: *P*<0.01; ***: *P*<0.001.

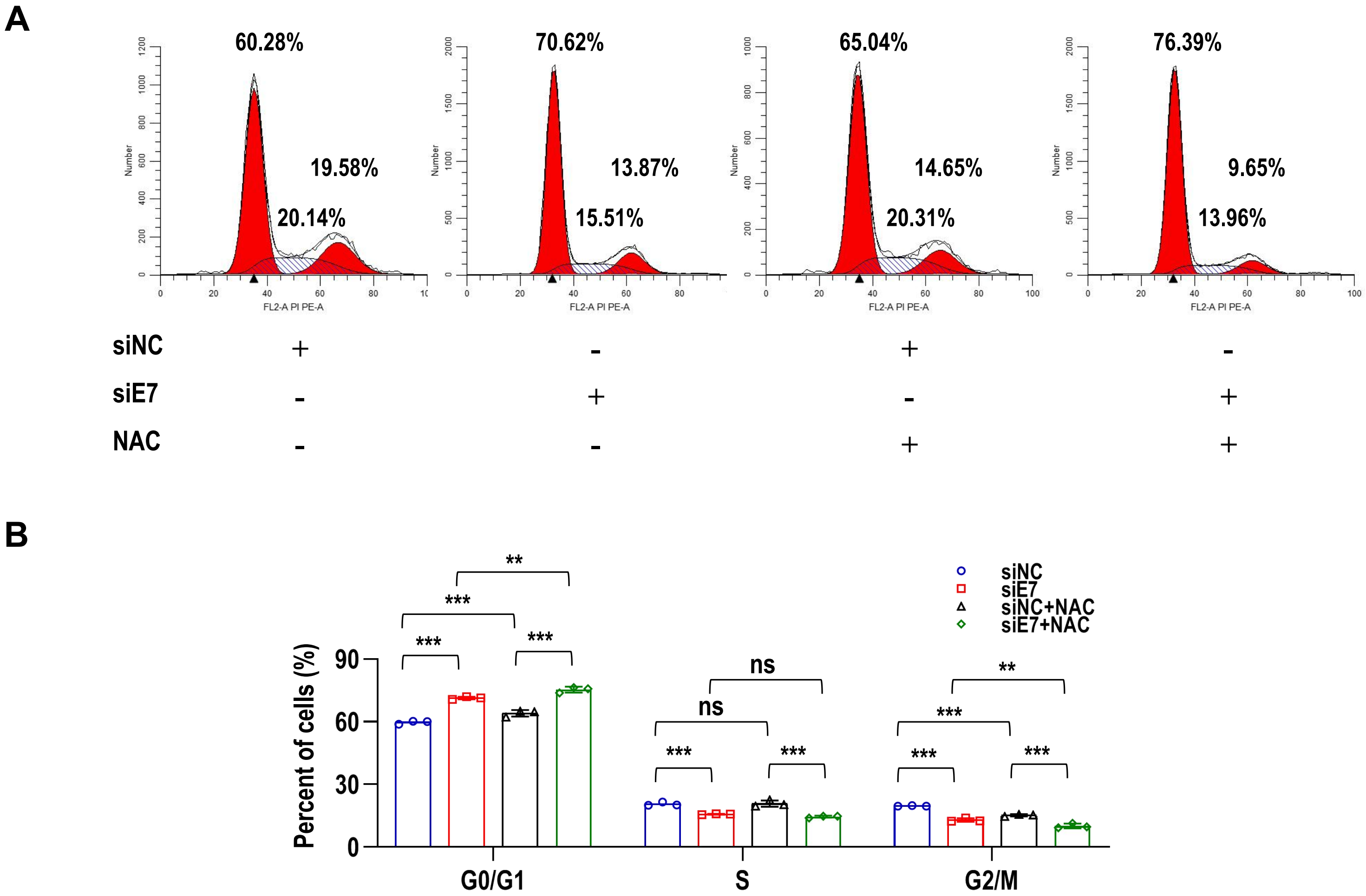

Figure S4. Effects of knocking down multiple splicing factors on the alternative splicing of Ki67 exon 7 in CAL 27 and SCC-9 cells. (**A-B**) Alternative splicing of Ki67 exon 7 were analyzed by RT-PCR. GAPDH served as the loading control. (**C-D**) The relative expression levels of splicing factors for checking knockdown efficiency. Data are mean ± SD, n = 3. *: *P*<0.05; **: *P*<0.01; ***: *P*<0.001.

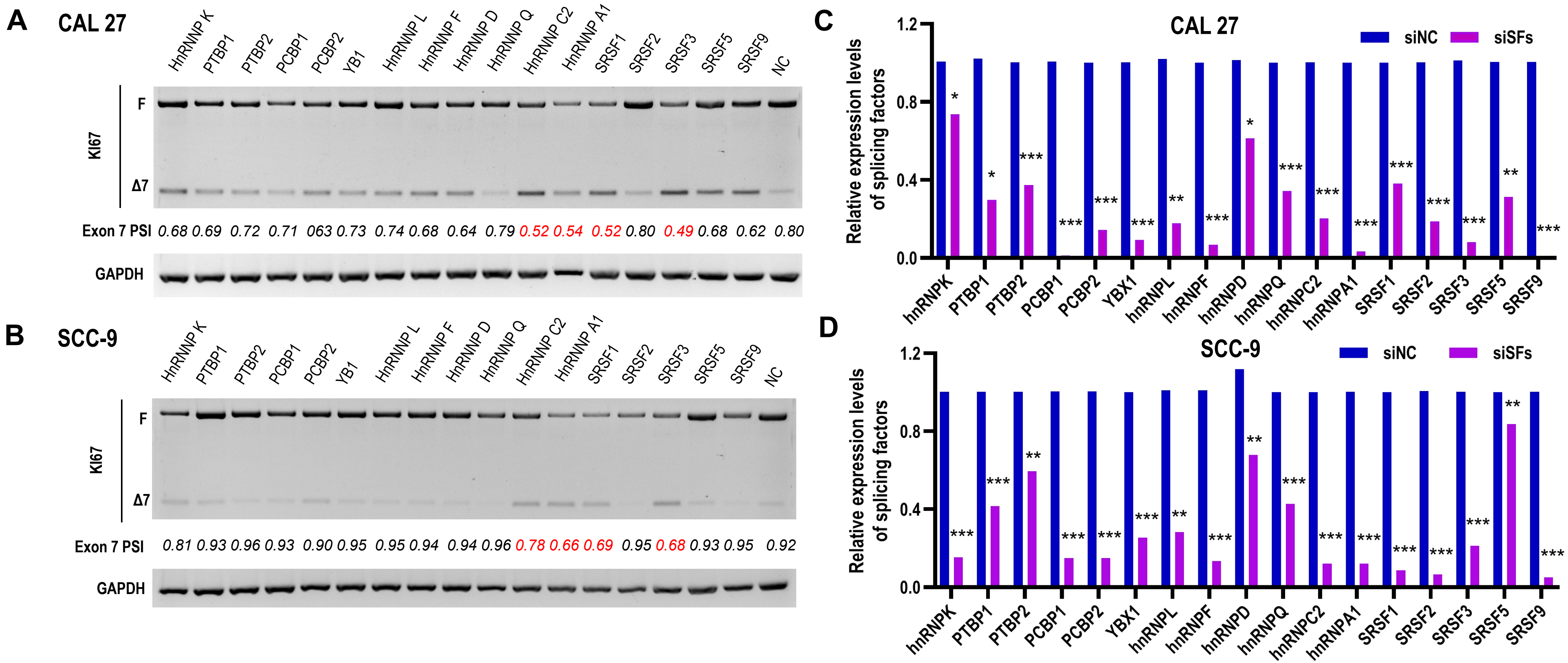

Figure S5. Representative images of HE staining in tumors dissected from nude mice. The scale bar represents 50 μm.

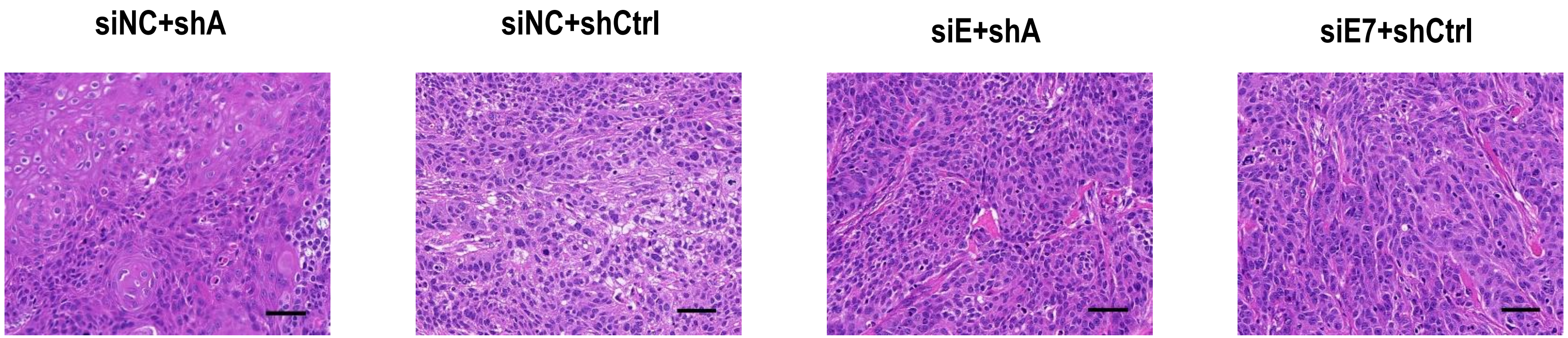

Supplement: Supplementary file 1 [file ijms-24-03872-s001.zip › Supplementary figures-IJMS.pdf]
