# Supplementary material for: SRSF3-Mediated Ki67 Exon 7-Inclusion Promotes Head and Neck Squamous Cell Carcinoma Progression via Repressing AKR1C2
Source: Int J Mol Sci. 2023 Feb 15;24(4):3872. doi: 10.3390/ijms24043872 (PMC9959251; doi:10.3390/ijms24043872)
Supplement: Supplementary file 1 [file ijms-24-03872-s001.zip › Supplementary tables-proof.pdf]

Table S1. Univariate and multivariate analysis of the prognostic value of the signature in terms of OS in HNSCC (TCGA).

Parameters	Univariate analysis				Multivariate analysis			
	P	HR	95%CI (lower/upper)		*P*	HR	95%CI (lower/upper)	
**Age(y)**<br>< 60 vs. ≥ 60	0.052	0.765	0.584	1.002	0.331	0.864	0.644	1.160
**Gender**<br>Male vs. Female	0.018	1.404	1.060	1.859	0.127	1.266	0.935	1.712
**Ki67 exon7 PSI**	0.003	4.554	1.661	12.490	0.016*	3.629	1.272	10.351
**Ki67 TPM**	0.822	1.001	0.990	1.012	0.865	0.999	0.988	1.011
**Radiation treatment**<br>No vs. Yes	0.001	1.579	1.210	2.060	0.001*	1.754	1.274	2.414
**Pharmaceutical drug adjuvant**<br>No vs. Yes	0.093	1.293	0.958	1.746	0.645	1.089	0.757	1.568
**T**<br>Stages I+II vs. Stages III+IV	< 0.001	0.554	0.411	0.746	0.003*	0.560	0.380	0.825
**N**<br>N0 vs. N1+2+3	< 0.001	0.554	0.405	0.758	0.001*	0.543	0.379	0.779
**Pathological stage**<br>Stages I+II vs. Stages III+IV	0.001	0.540	0.373	0.783	0.468	0.821	0.481	1.399

*: $p < 0.05$.

Table S2. siRNA sequences and references.

Target	siRNA sequences 5'-3'	References
HNRNPK	CCAACAUUCCUCUGCUUCA	--
PTBP1	UGACAAGAGCCGUGACUAC	[1]
PTBP2	GCTGTACCCTAAGGATTGATT	--
PCBP1	GAACCAGGUGGCAAGACAA	[2]
PCBP2	GGCCUAUACCAUUCAAGGA	[3]
YBX1	GGUCAUCGCAACGAAGGUU	[4]
HNRNPL	CUACGAUGACCCGCACAAA	[5, 6]
HNRNPF	GGAAUGUAUGACCACAGAUAC	--
HNRNPD	AGACUGCACUCUUGAAGUUA	[7]
HNRNPQ	GGACUUACAGCUAAACAAC	[8]
HNRNPC2	GAGATGTACGGGTCAGTAA	--
HNRNPA1	GUGGUAAUGAGAGAUCCAA	--
SRSF1	ACGAUUGCCGCAUCUACGU	[9]
SRSF2	CCAUCGAAGAAGAGUCCUU	--
SRSF3	AGAGCUAGAUGGAAGAACA	--
SRSF5	AAUUUAUCCUCAAGAGUCAGCUG	--
SRSF9	GGAAUAUGCCCUGCGUAAA	--

1. Guo, J.; Jia, J.; Jia, R. Ptbp1 and Ptbp2 Impaired Autoregulation of Srsf3 in Cancer Cells. *Sci Rep* **2015**, *5,* 14548. https://doi.org/10.1038/srep14548.
2. Wang, X.; Guo, J.; Che, X.; Jia, R. Pcbp1 Inhibits the Expression of Oncogenic Stat3 Isoform by Targeting Alternative Splicing of Stat3 Exon 23. *Int J Biol Sci* **2019**, *15,* 1177-86. https://doi.org/10.7150/ijbs.33103.
3. Ghosh, D.; Srivastava, G.P.; Xu, D.; Schulz, L.C.; Roberts, R.M. A Link between Sin1 (Mapkap1) and Poly(Rc) Binding Protein 2 (Pcbp2) in Counteracting Environmental Stress. *Proceedings of the National Academy of Sciences of the United States of America* **2008**, *105,* 11673-78. https://doi.org/10.1073/pnas.0803182105.
4. Jia, R.; Liu, X.; Tao, M.; Kruhlak, M.; Guo, M.; Meyers, C.; Baker, C.C.; Zheng, Z.M. Control of the Papillomavirus Early-to-Late Switch by Differentially Expressed Srp20. *JOURNAL OF VIROLOGY* **2009**, *83,* 167-80. https://doi.org/10.1128/JVI.01719-08.
5. Jia, R.; Zhang, S.; Liu, M.; Zhang, Y.; Liu, Y.; Fan, M.; Guo, J. Hnrnp L Is Important for the Expression of Oncogene Srsf3 and Oncogenic Potential of Oral Squamous Cell Carcinoma Cells. *ScientIF-ic Reports* **2016**, *6,* 35976. https://doi.org/10.1038/srep35976.
6. Xu, L.; Shen, J.; Jia, J.; Jia, R. Inclusion of Hnrnp L Alternative Exon 7 Is Associated with Good Prognosis and Inhibited by Oncogene Srsf3 in Head and Neck Squamous Cell Carcinoma. *Biomed Res Int* **2019**, *2019,* 9612425. https://doi.org/10.1155/2019/9612425.
7. Lund, N.; Milev, M.P.; Wong, R.; Sanmuganantham, T.; Woolaway, K.; Chabot, B.; Abou Elela, S.; Mouland, A.J.; Cochrane, A. Differential Effects of Hnrnp D/Auf1 Isoforms on Hiv-1 Gene Expression. *Nucleic Acids Res* **2012**, *40,* 3663-75. https://doi.org/10.1093/nar/gkr1238.
8. Chen, Y.; Tian, D.; Ku, L.; Osterhout, D.J.; Feng, Y. The Selective Rna-Binding Protein Quaking I (Qki) Is Necessary and Sufficient for Promoting Oligodendroglia Differentiation. *J Biol Chem*

2007*, 282,* 23553-60. https://doi.org/10.1074/jbc.M702045200.

9. Chen, Z.; Ma, X.; Zhang, J.; Hu, J.; Gorczynski, R.M. Alternative Splicing of Cd200 Is Regulated by an Exonic Splicing Enhancer and Sf2/Asf. *Nucleic Acids Res* **2010***, 38,* 6684-96. https://doi.org/10.1093/nar/gkq554.

Table S3. Primers for plasmids construction.

Target	Forward primers (5'-3')	Reverse primers (5'-3')
Ki67 primers 1	ACCCTGCGACTCTCCACAGTC	TCTGTCAAATATCTTCACTGTCCCTATG
Ki67 primers 2	GGATCTATTTCCGGTGAATTCCCTTCCCCTACGGATTATACTCAAC	GCTACCAGTTACACTTGCTGCTG
Minigene overlap primer 1	CAGCACGTCGTGTCTCAAGATC	
Minigene overlap primer 2		CATCAAATAGTTCAGGTCTTAGGTGC
Minigene-wt	TCGAGCTCAAGCTTCGAATTCCAGCACGTCGTGTCTCAAGATC	ATGGTGGCGACCGGTGGATCCCATCAAATAGTTCAGGTCTTAGGTGC
ΔE7F1	AATTCATTGCCAAGATGAGAAAGAAATTTCCAGCGTTAAATTAGT	ACTAATTTAACGCTGGAAATTTCTTTCTCATCTTGGCAATGAATT
ΔE7F2	CATTTCTGGGGATTTTAAAGAAAACAGAGAAAGAAAGTGCTGATG	CATCAGCACTTTCTTTCTCTGTTTTCTTTAAAATCCCCAGAAATG
ΔE7F3	GGATTACAAACTGATTACGCAACTATGAGCCGGCTAAAATGAAG	CTTCATTTTAGCCGGCTCATAGTTGCGTAATCAGTTTGTAATCC
ΔE7F4	GCGCCAGCTTTCCTCTCTATAAGATGCAGCTGACTCTGCC	GGCAGAGTCAGCTGCATCTTATAGAGAGGAAAGCTGGCGC
ΔE7F5	CGAACACCAGCTAAAGTTGAAGGATTCCATTAGTAAGTTTATTTAATTGT	ACAATTAAATAAACTTACTAATGGAATCCTTCAACTTTAGCTGGTGTTCG
ΔE7F4-1	GCGCCAGCTTTCCTCTCTATGACCTGTATACTACTGGTAGAAGAGAA	TTCTCTTCTACCAGTAGTATACAGGTCATAGAGAGGAAAGCTGGCGC
ΔE7F4-2	CAAAATTCTCCACAAAAACATAAGTTCAAGGCTGGTGATAAAACTC	GAGTTTTATCACCAGCCTTGAACTTATGTTTTTGTGGAGAATTTTG
ΔE7F4-3	ATCTGTGAATCTGGGTAAAAGTGAAAGATGCAGCTGACTCTGCC	GGCAGAGTCAGCTGCATCTTTCACTTTTACCCAGATTCACAGAT
Mt1	TCTGGGTAAAAGTGAACGATACTACGCTGGTGATAAAACT	AGTTTTATCACCAGCGTAGTATCGTTCACTTTTACCCAGA
Mt2	AGTGAAGGCTTCAAGGATCGAGTTTAAACTCTTACTCCCA	TGGGAGTAAGAGTTTAAACTCGATCCTTGAAGCCTTCACT
Mt3	TCAAGGCTGGTGATAATAGTGTAAGTCCCAGGAAGCTTTC	GAAAGCTTCCTGGGACTTACACTATTATCACCAGCCTTGA
Mt4	TGATAAAACTCTTACTGCGACGTACCTTTCAACTAGAAAT	ATTTCTAGTTGAAAGGTACGTCGCAGTAAGAGTTTTATCA
Mt5	CTTACTCCCAGGAAGCATACTAGTTGAAATCGAACACCAG	CTGGTGTTCGATTTCAACTAGTATGCTTCCTGGGAGTAAG
M6	GGAAGCTTTCAACTAGTATTGGTAGACCAGCTAAAGTTGA	TCAACTTTAGCTGGTCTACCAATACTAGTTGAAAGCTTCC
Mt7	AACTAGAAATCGAACAGCTGGTTATGTTGAAGATGCAGCT	AGCTGCATCTTCAACATAACCAGCTGTTCGATTTCTAGTT

Table S4. Primers used in RT-PCR.

Target	Forward primers (5’-3’)	Reverse primers (5’-3’)
Ki67	GAACAGCCTCAACCATCAGGA	GCCACTCTTTCTCCCTCCTCTC
Ki67 exon 7 skipping	TCAGAATGGAAGGAAGTCAACTGA	CATCAAATAGTTCAGGTCTTAGGTGC
Ki67 exon 7 skipping in minigene	CAGCACGTCGTGTCTCAAGATC	GCTCCTCGCCCTTGCTCACCA
GAPDH	GAAGGTGAAGGTCGGAGTC	GAAGATGGTGATGGGATTTC

Table S5. Primers used in qRT-PCR.

Gene	Forward primers (5'-3')	Reverse primers (5'-3')
CD82	GGAAGAGGACAACAGCCTTTCTG	ATGATGCCCAGGTTCTCCTGCA
ITGA2	CCTACAATGTTGGTCTCCCAGA	AGTAACCAGTTGCCTTTTGGATT
ITGA3	TGTGGCTTGGAGTGACTGTG	TCATTGCCTCGCACGTAGC
ITGA6	CAGTGGAGCCGTGGTTTTG	CCACCGCCACATCATAGCC
ITGB2	AGTCACCTACGACTCCTTCTGC	CAAACGACTGCTCCTGGATGCA
ITGB3	CATGGATTCCAGCAATGTCCTCC	TTGAGGCAGGTGGCATTGAAGG
IL24	CTTCTCTGGAGCCAGGTATCAG	GGCACTCGTGATGTTATCCTGAG
IL1R2	GGCTATTACCGCTGTGTCCTGA	GAGAAGCTGATATGGTCTTGAGG
IL23A	GAGCCTTCTCTGCTCCCTGATA	GACTGAGGCTTGGAATCTGCTG
IL6ST	CACCCTGTATCACAGACTGGCA	TTCAGGGCTTCCTGGTCCATCA
WNT7A	AGGAGAAGGCTCACAAATGGGC	CGGCAATGATGGCGTAGGTGAA
WNT7B	AGAAGACCGTCTTCGGGCAAGA	AGTTGCTCAGGTTCCCTTGGCT
TGFB2	AAGAAGCGTGCTTTGGATGCGG	ATGCTCCAGCACAGAAGTTGGC
LAMA3	TAGAGGAAGCCTCTGACACAGG	CCGATAGTATCCAGGGCTACAAC
LAMC2	TACAGAGCTGGAAGGCAGGATG	GTTCTCTTGGCTCCTCACCTTG
CSF2	GGAGCATGTGAATGCCATCCAG	CTGGAGGTCAAACATTTCTGAGAT
TGM2	TGTGGCACCAAGTACCTGCTCA	GCACCTTGATGAGGTTGGACTC
GCLC	ATGTGGACACCCGATGCAGTATT	TGTCTTGCTTGTAGTCAGGATGGTTT
GCLM	GCCACCAGATTTGACTGCCTTT	CAGGGATGCTTTCTTGAAGAGCTT
GSR	TATGTGAGCCGCCTGAATGCCA	CACTGACCTCTATTGTGGGCTTG
PRDX4	CGCTTTTGGCGACAGACTTGAAG	CCAAGTCCTCCTTGTCTTCGAG
SOD2	GGAAGCCATCAAACGTGACTT	CCCGTTCCTTATTGAAACCAAGC
AKR1C1	GCAAGTCAAAAGACATTGTTCTGG	TTGCCAAGGCACAAAGGACTGG
AKR1C2	CAGTGGATCTCTGTGCCACATG	CTGGTTGCAGACAGGCTTGTAC
AKR1C3	CCGAAGCAAGATTGCAGATGGC	GTGAGTTTTCCAAGGCTGGTCG
